# Interdigitated immunoglobulin arrays form the hyperstable surface layer of the extremophilic bacterium *Deinococcus radiodurans*

**DOI:** 10.1073/pnas.2215808120

**Published:** 2023-04-12

**Authors:** Andriko von Kügelgen, Sofie van Dorst, Keitaro Yamashita, Danielle L. Sexton, Elitza I. Tocheva, Garib Murshudov, Vikram Alva, Tanmay A. M. Bharat

**Affiliations:** ^a^Structural Studies Division, MRC Laboratory of Molecular Biology, Cambridge CB2 0QH, United Kingdom; ^b^Sir William Dunn School of Pathology, University of Oxford, Oxford OX1 3RE, United Kingdom; ^c^Department of Microbiology and Immunology, University of British Columbia, Vancouver, BC V6T 1Z3, Canada; ^d^Department of Protein Evolution, Max Planck Institute for Biology Tübingen, Tübingen 72076, Germany

**Keywords:** cell surface, *Deinococcus radiodurans*, cryo-EM, surface layer, immunoglobulin

## Abstract

*Deinococcus radiodurans* is an atypical diderm bacterium with a remarkable ability to tolerate various environmental stresses, due in part to its complex cell envelope encapsulated within a hyperstable surface layer (S-layer). Despite decades of research on this cell envelope, atomic structural details of the S-layer have remained obscure. In this study, we report the electron cryomicroscopy structure of the *D. radiodurans* S-layer, showing how it is formed by the Hexagonally Packed Intermediate-layer (HPI) protein arranged in a planar hexagonal lattice. The HPI protein forms an array of immunoglobulin-like folds within the S-layer, with each monomer extending into the adjacent hexamer, resulting in a highly interconnected, stable, sheet-like arrangement. Using electron cryotomography and subtomogram averaging from focused ion beam-milled *D. radiodurans* cells, we have obtained a structure of the cellular S-layer, showing how this HPI S-layer coats native membranes on the surface of cells. Our S-layer structure from the diderm bacterium *D. radiodurans* shows similarities to immunoglobulin-like domain-containing S-layers from monoderm bacteria and archaea, highlighting common features in cell surface organization across different domains of life, with connotations on the evolution of immunoglobulin-based molecular recognition systems in eukaryotes.

## Significance

*Deinococcus radiodurans* is a bacterium with a remarkable ability to tolerate various environmental stresses, partly because of a hyperstable proteinaceous surface layer (S-layer) that forms the outermost layer of its cell envelope. In this study, we report the atomic structure of the S-layer of *D. radiodurans* and show how it is formed by the self-assembly of a protein containing immunoglobulin domains in a highly interconnected arrangement. Our results solve a long-standing mystery about the cell surface of *D. radiodurans* and about the identity of the protein that forms the S-layer. Furthermore, this S-layer shares similarities with immunoglobulin domain-containing S-layers from distantly related bacteria and archaea, providing clues for unraveling the evolution of immunoglobulin-based molecular recognition systems in eukaryotes.

*Deinococcus radiodurans* is an atypical diderm bacterium with a remarkable ability to tolerate various environmental stresses, including nuclear radiation, extreme temperatures, vacuum, oxidation, and desiccation (1, 2). In fact, it can tolerate acute doses of ionizing γ-radiation up to 5,000 Grays (Gy) without loss of viability (2). In comparison, doses of 5 Gy are lethal to humans and 200 to 800 Gy to *Escherichia coli* (3). Because of its extreme tolerance to radiation and desiccation, *D. radiodurans* can survive not only under extreme conditions on Earth but also for years in outer space (4). Therefore, *D. radiodurans* is of great interest for biotechnological applications such as bioremediation of radioactive waste. The biochemical mechanisms underlying the hyperstability of *D. radiodurans* are not yet fully understood. However, its efficient mechanisms for repairing damaged DNA (5), its molecular machinery for preventing oxidative protein damage (6, 7), and its complex cell envelope are thought to be important factors promoting hyperstability (8–12).

*D. radiodurans* is the prototypical species of the evolutionarily deep-branching genus Deinococcus, which is considered to represent an intermediate in the diderm–monoderm transition exhibiting features of both Gram-negative and Gram-positive bacteria (2). While *D. radiodurans* stains Gram-positive because it contains a thick peptidoglycan (PG) layer covering the inner membrane (IM), its cell envelope resembles that of Gram-negative bacteria because it has a periplasmic space and an outer membrane (OM) (8–10). In addition, the OM of *D. radiodurans* is covered by a carbohydrate-coated hexagonal surface layer (S-layer) (13), a proteinaceous, paracrystalline layer commonly found on the surface of archaea and bacteria (14). In general, S-layers are involved in critical cellular functions such as maintenance of cell shape, protection from phages, biomineralization, cell adhesion, molecular recognition, and induction of virulence (15). The S-layer in *D. radiodurans* is thought to be composed entirely of the Hexagonally Packed Intermediate-layer (HPI) surface protein and exhibits impressive hyperstability at high temperatures (>80 °C) even in the presence of strong detergents such as sodium dodecyl sulfate (SDS) and denaturing agents such as urea (13, 16). While several pioneering studies have revealed the molecular envelope formed by the S-layer of *D. radiodurans* using three-dimensional electron microscopy (EM) (9, 11, 13, 17–22), the atomic structure of the S-layer, the principles governing its assembly, and its association with other components of the cell envelope remain elusive.

In this study, we report the cryogenic electron microscopy (cryo-EM) structure of the S-layer of *D. radiodurans.* Our structure confirms that this S-layer consists exclusively of the HPI protein arranged in a hexagonal lattice, with no density observed for other proteins. The HPI protein within the S-layer is arranged as an interdigitated array of immunoglobulin-like (Ig-like) domains, with interhexameric linkages formed by the exchange of one Ig-like domain with the adjacent hexameric center, resulting in a highly interconnected sheath-like arrangement. Cryo-electron tomography (cryo-ET) of focused ion beam (FIB)-milled cells supports our cryo-EM structure and confirms the organization of HPI proteins within the cellular *D. radiodurans* S-layer. Comparing the S-layer structure of *D. radiodurans* with that of Gram-positive bacteria such as *Bacillus anthracis* (23) and *Geobacillus stearothermophilus* (24, 25), and the archaeon *Haloferax volcanii* (26), we find that Ig-like domains are a common feature of all these S-layer proteins (SLPs). Our observations suggest a common mechanism for cell surface organization shared across different domains of life, with implications for understanding the evolution of eukaryotic molecular recognition systems based on immunoglobulin domains.

## Results

### Cryo-EM Structure of the *D. radiodurans* HPI S-Layer.

**Fig. 1.**
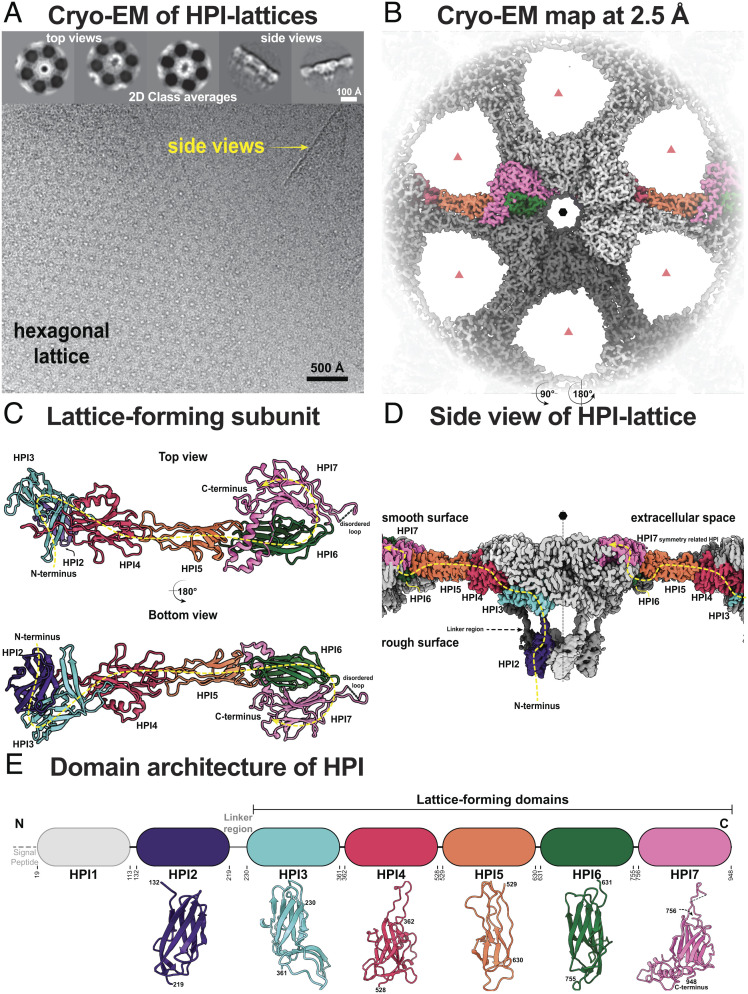
Cryo-EM reconstruction of *D. radiodurans* S-layer. (*A*) Cryo-EM images of purified *D. radiodurans* cell envelopes show hexagonal sheets of S-layer, in line with previous reports. *Insets*—characteristic top and side views observed in class averages. (*B*) Density map of the S-layer is shown from the top, with two (twofold) symmetry-related copies of SLP monomers shown in full color. The resolution of the map is 2.5 Å (*SI Appendix*, Fig. S1). The nearly hexameric (black hexagon) and trimeric symmetry (light red triangles) axes are marked. During cryo-EM refinement, only sixfold symmetry was imposed. (*C*) The S-layer is made up of repeating copies of a single SLP called HPI, shown as a ribbon diagram. The path of one monomer is shown with a dotted yellow line. (*D*) The HPI S-layer cryo-EM map shown in an orthogonal orientation compared to panel *B*, a view along the lattice. (*E*) Schematic cartoon of the HPI protein sequence together with resolved cryo-EM structures of individual HPI domains. HPI1 is not resolved in our cryo-EM map and is likely involved in anchoring the lattice to the cell.

To elucidate the organization of the cell envelope in *D. radiodurans*, we proceeded to solve the atomic structure of the purified S-layer. For this purpose, we isolated native S-layer (*Materials and Methods*) by adapting a previously described protocol that employed SDS solubilization (8, 13). Images of the isolated S-layer revealed top and side views of S-layer-like two-dimensional sheets displaying a characteristic planar hexagonal symmetry (Fig. 1*A*). We used a previously described single-particle cryo-EM data analysis workflow (26) to resolve a global 2.5 Å-resolution structure of the S-layer within the planar two-dimensional lattice (Fig. 1 *B*–*D* and *SI Appendix*, Fig. S1 and Table S1). The 2.5 Å-resolution map allowed us to build an atomic model of the *D. radiodurans* S-layer (Fig. 1 *C*–*E* and Movie S1).

**Fig. 2.**
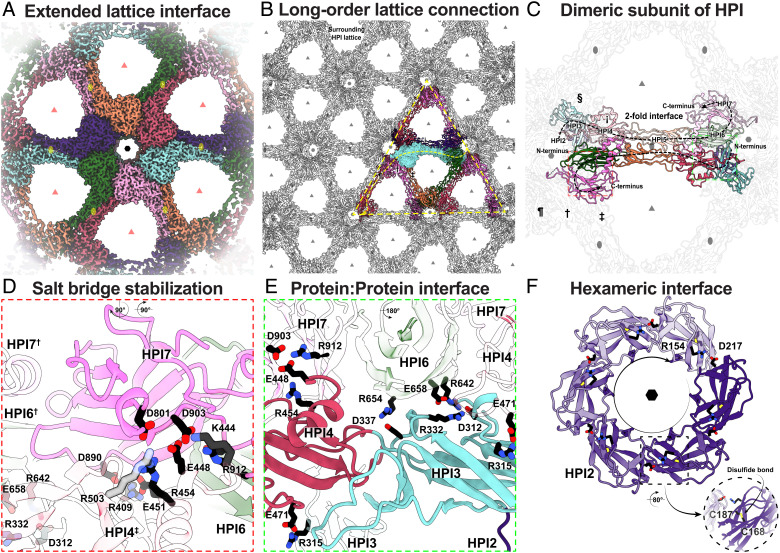
HPI Ig-like domains form a highly interconnected lattice stabilized by multiple salt bridges. (*A*) Cryo-EM density of an HPI hexamer. Different HPI monomers are colored differently with the hexameric, trimeric, and dimeric axes marked by a black hexagon, light red triangles, and yellow ovals, respectively. (*B*) Each HPI monomer (cyan) interacts tightly with nine other monomers, spanning six hexamers of the lattice, leading to a highly interconnected S-layer. (*C*) Each HPI monomer swaps into the adjoining hexamer through the extended HPI5 domain. At the central hexameric interface, symmetry-related HPI subunits are labeled clockwise with (‡, †, ¶, §, and ¡). The lattice is stabilized by multiple salt bridges, shown in enlarged views marked with a (*D*) red box, and (*E*) green box. (*F*) The hexameric HPI2 domain is also stabilized by several salt bridges and a disulfide bond in the HPI2 domain (*Inset*). For lattice-wide salt bridge networks, see *SI Appendix*, Fig. S4 *B*–*D*.

The atomic model of the S-layer shows that only the HPI protein forms the S-layer, with no additional density observed in the map (Fig. 1 *B* and *D* and *SI Appendix*, Fig. S1), consistent with previous reports (8, 9, 11–13, 20–22). At the sequence level, the HPI protein consists of seven β-strand-rich immunoglobulin (Ig)-like domains (HPI1 to HPI7 from hereon, Fig. 1*E* and *SI Appendix*, Fig. S2), six of which (HPI2–HPI7) were resolved in our cryo-EM map and revealed an extended arrangement of the HPI protein. The segment comprising the HPI1 domain (residues 19 to 131) was not resolved in our structure, suggesting flexibility in its position with respect to the S-layer lattice. To obtain structural information on HPI1 and its connectivity to HPI2, we used AlphaFold2 (27) to build a structural model of full-length HPI including the unresolved segment in our structure. The predicted model shows that HPI1 also has an Ig-like fold and is likely connected to HPI2 through a long, disordered linker (residues 114 to 131) (*SI Appendix*, Fig. S3). The first experimentally resolved domain, HPI2 (residues 132 to 219), is connected to HPI3 through a long, extended linker containing residues 220 to 229 (Fig. 1*D*). HPI3–HPI7 form a planar sheet (Figs.1*D* and 2 and *SI Appendix*, Fig. S1) and exhibit high sequence variability relative to each other and to HPI1 and HPI2 (Fig. 1*E*). Despite this variability, all seven Ig-like domains of HPI are structurally similar and resemble the topology group (T-group) “immunoglobulin/fibronectin type III/E set domains/PapD-like” in the Evolutionary Classification of Protein Domains (ECOD) database (28).

The domains of HPI exhibit a bipartite arrangement, with a long linker (residues 220 to 229, Fig. 1*D*) separating HPI1-HPI2 from HPI3-HPI7, suggesting that while the latter domains form the canopy of the S-layer, the former ones form the OM-anchoring stalk of the S-layer (14). Indeed, HPI is predicted to possess an N-terminal lipoprotein signal peptide with a canonical, four-residue-long lipobox motif containing a conserved cysteine residue at the last position. Lipobox-containing lipoproteins are widespread in bacteria, and they have been previously described to be anchored to the OM by posttranslational lipid modification of the conserved cysteine residue that forms the N terminus of the mature protein after cleavage of the signal peptide (29). Indeed, a conserved cysteine is the first residue in the mature HPI protein, suggesting that HPI may be anchored to the OM by lipidation (*SI Appendix*, Fig. S2).

### HPI Monomers from an Interdigitated Array in the *D. radiodurans* S-Layer.

The extended monomers of HPI are packed in a sheet as an interdigitated array (Fig. 2 *A*–*C* and Movie S1). Each monomer spans two hexamers of the S-layer lattice via HPI5, which is arranged as a twofold symmetric bridge linking adjoining hexamers (Fig. 2*C*). This extended arrangement partly explains the exceptional stability of the *D. radiodurans* S-layer, since there are large protein:protein interfaces making up the lattice (Fig. 2*C*). Each hexamer of the S-layer lattice itself consists of two stacked tiers, the first formed by HPI3 and HPI4, connected by the HPI5 dimeric bridge, and the second (top) formed by HPI6 and HPI7 (*SI Appendix*, Fig. S4*A*).

This extended arrangement of HPI monomers results in two major types of pores observed in the lattice (Fig. 2 *A* and *B*). The hexameric pore is relatively large (~33 Å) compared to the S-layer structure reported for the diderm Gram-negative *Caulobacter crescentus* (20 Å, *SI Appendix*, Fig. S5 *A* and *B*). There are additional gaps between the *D. radiodurans* S-layer hexamers that are even larger (~76 Å), resulting in a relatively holey structure, suggesting that this S-layer is not acting to occlude small molecules from the cell surface (Fig. 2 *A* and *B*). In the Gram-negative S-layer lattice of *C. crescentus*, the hexameric, trimeric, and dimeric interfaces that make up the lattice are lined with Ca^2+^ ions, which have been shown to be essential for lattice assembly and stabilization (30–32). In the same vein, some putative cation-binding sites are also seen in the HPI lattice (*SI Appendix*, Fig. S5 *C* and *D*), which may play a role in stabilizing the lattice. Additionally, the S-layer is stabilized by many salt bridges that form an extensive network through the HPI lattice (Fig. 2 *D*–*F* and *SI Appendix*, Fig. S4 *B*–*D*). This network of salt bridges likely plays a key role in the observed hyperstability of the *D. radiodurans* S-layer. Finally, there are two cysteine residues in close proximity (C554 and C666) between HPI5 and HPI6 of each monomer (*SI Appendix*, Figs. S2 and S5*H*). Although densities connecting these residues were not observed in the map, they could form a putative disulfide bridge connecting HPI5 to HPI6, further interconnecting the lattice (Fig. 2 *D*–*F*).

### Ig-Like Arrays Are Observed in S-Layers across Bacterial and Archaeal Phyla.

**Fig. 3.**
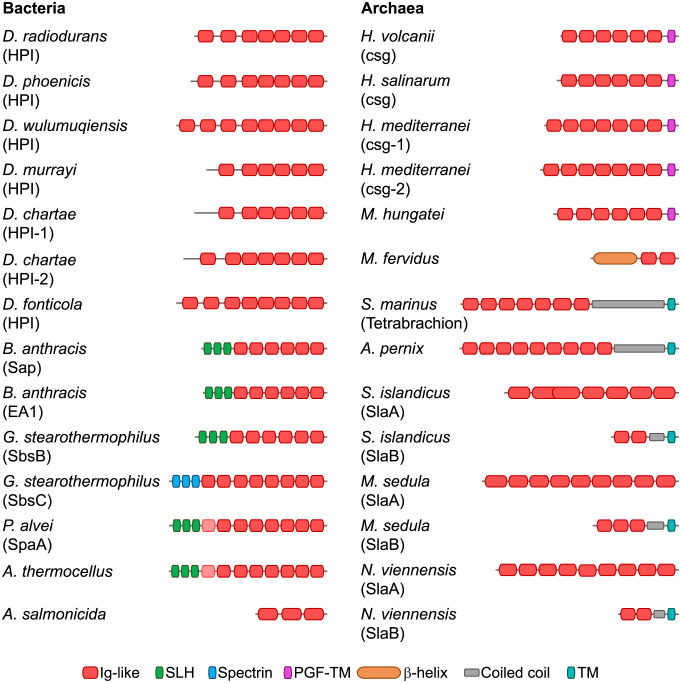
Domain organization of prokaryotic Ig-like domain-containing SLPs. The Ig-like domain arrays observed in the *D. radiodurans* HPI protein are found in many bacterial and archaeal SLPs. However, these SLPs employ different anchoring mechanisms; for example, while many Gram-positive bacterial SLPs have N-terminal PG-binding SLH domains, many archaeal SLPs have a C-terminal TM helix. In addition to Ig-like and anchoring domains, some archaeal SLPs also contain other domains, such as ß-helical and coiled-coil segments. The first Ig-like domain of SLPs from *Paenibacillus alvei* and *Acetivibrio thermocellus* is highly divergent and is therefore colored in a lighter shade of red. In SlpA of **Sulfolobus* islandicus*, the third Ig-like domain is nested within the second Ig-like domain. Some organisms, such as *B. anthracis*, have been found to exhibit two different S-layers composed of two different proteins (Sap and EA1). In fact, many archaea (e.g., *Haloferax mediterranei*) and bacteria (*D. chartae*) have more than one predicted SLP and likely exhibit different S-layers. While most S-layers are assembled by the polymerization of one protein, the S-layer of organisms such as **Sulfolobus* islandicus* and **Staphylothermus* marinus* is composed of two different protein chains. The two protein chains of the S-layer of **Staphylothermus* marinus* are encoded by a single gene and are obtained through proteolytic cleavage. Accession details for the shown proteins are provided in *SI Appendix*, Table S2.

To investigate whether HPI-like S-layers occur in other bacteria, particularly in the phylum Deinococcota, we searched for homologs of HPI in the National Center for Biotechnology Information (NCBI) nonredundant (nr) protein sequence database using the Basic Local Alignment Search Tool (BLAST). The search found about 20 homologs, mainly from bacteria of the order Deinococcales, including *Deinococcus wulumuqiensis*, *Deinococcus phoenicis*, *Deinococcus murrayi*, *Deinococcus fonticola*, and *Deinobacterium chartae*, which has two paralogs (Fig. 3 and *SI Appendix*, Fig. S2 and Table S2). The search also identified a homolog in *Thermus thermophilus* (NCBI ID BDG20071.1), but it is unknown whether it forms an S-layer in vivo. The sparse occurrence of HPI suggests that HPI-like S-layers may not be widespread in the phylum Deinococcota or that the sequences of many HPI proteins have diverged considerably, making their detection difficult.

The deinococcal HPI proteins identified in the search are quite conserved and share pairwise sequence identities of 25-88% with *D. radiodurans* HPI. Like *D. radiodurans* HPI, most homologs contain seven consecutive Ig-like domains, with some possessing additional N- or C-terminal Ig-like domains or lacking an N-terminal domain (Fig. 3). For example, compared to *D. radiodurans*, HPI of *D. murrayi* lacks the first Ig-like domain, whereas HPI of *D. wulumuqiensis* has an additional N-terminal Ig-like domain, suggesting that the length of the stalk region connecting the OM to the canopy of the S-layer may vary (Fig. 3). On the contrary, the HPI proteins of *D. fonticola* and *D. chartae* contain an additional Ig-like domain at their C-termini. Furthermore, all HPI homologs are predicted to contain a lipoprotein signal peptide and an invariant N-terminal cysteine residue as the first residue in the mature protein, strongly suggesting that HPI is anchored to the OM through lipidation. Lastly, the salt bridges and the putative disulfide bridge between HPI5 and HPI6 mentioned above and a disulfide bridge in HPI2 are highly conserved in all homologs (*SI Appendix*, Fig. S2), indicating that all HPI S-layers are probably hyperstable, in the same manner as the HPI S-layer of *D. radiodurans*.

In addition to HPI, arrays of consecutive Ig-like domains have also been structurally characterized in the SLPs of the archaeon *H. volcanii* (csg; PDB 7PTR) (26) and the Gram-positive bacteria *B. anthracis* (Sap; PDB 6QX4) and *G. stearothermophilus* (SbsB; PDB 4AQ1 and SbsC; PDB 4UIC) (23, 24). The Ig-like domains of these proteins have a fold similar to that of the Ig-like domains of HPI and have been classified into the aforementioned T-group “immunoglobulin/fibronectin type III/E set domains/PapD-like” in the ECOD database (*SI Appendix*, Fig. S3). Additionally, we analyzed the domain composition of many experimentally characterized SLPs using sensitive sequence searches and structural modeling with AlphaFold2 and found that Ig-like domains are widespread in prokaryotic SLPs (*Materials and Methods* and Fig. 3). These SLPs often exhibit different membrane-anchoring mechanisms and occasionally possess additional non-Ig-like domains. For example, many Gram-positive bacterial SLPs contain N-terminal PG-binding S-layer homology (SLH) domains, whereas archaeal SLPs are anchored by lipidation of their C-terminal end or through a C-terminal transmembrane (TM) helix (14). Finally, although some prokaryotic SLPs lacking Ig-like domains have also been structurally characterized, such as the SLPs of the archaeon *Methanosarcina acetivorans* (33), the Gram-positive bacterium *Clostridioides difficile* (34), and the Gram-negative bacterium *C. crescentus* (30–32), Ig-like arrays appear to be the most common protein arrangement in S-layers.

### In Situ Organization of the S-Layer.

**Fig. 4.**
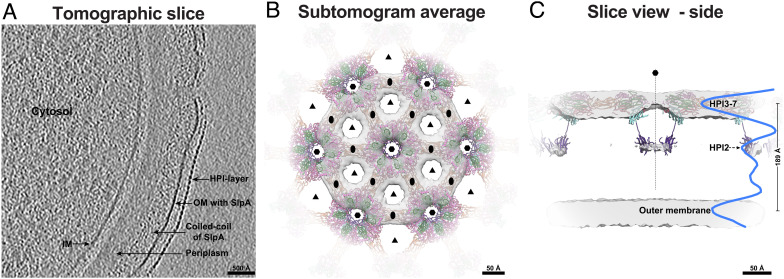
Imaging FIB-milled *D. radiodurans* cells confirms in vitro structural data. (*A*) A tomographic slice through a FIB-milled *D. radiodurans* cell (10) shows the expected density layers on the cell surface corresponding to the S-layer, OM, PG, and IM. (*B*) Subtomogram averaging of the S-layer confirms the hexagonal arrangement of the S-layer. Docking of the atomic model (ribbon) into the map (gray isosurface) further shows that HPI is the only protein in the S-layer. (*C*) An orthogonal view of the S-layer subtomogram average shows weak density for HPI2 and an additional peak that might correspond to HPI1; the density profile is shown in blue.

To verify whether our structure from purified S-layers is consistent with the native assembly of HPI proteins, we collected cryotomograms of whole cells. Since *D. radiodurans* cells are ~4 μm in diameter, we first vitrified them on Finder grids and then generated 200-nm thick lamellae using cryo-FIB milling (10). Cryotomograms revealed that the in situ appearance of the S-layer is same as that of the purified S-layer (Fig. 4*A*), indicating that the native S-layer has the same arrangement as our purified sample.

Next, we used subtomogram averaging of the S-layer lattice using previously described methods (32, 35, 36), producing a 26 Å-resolution reconstruction of the cellular S-layer (Fig. 4 *B* and *C* and *SI Appendix*, Fig. S6). Although limited in resolution, our subtomogram averaging agrees with the atomic structure of the HPI S-layer and shows that both are organized in a hexagonal array with the same lattice spacing (Fig. 4 *B* and *C*). The S-layer is seen as large patches, punctuated with discontinuities, coating the cell surface, as reported previously (10, 37). The multilayered arrangement of the S-layer was also confirmed by our subtomogram average, with densities observed for both the canopy (HPI3-7) and the base (HPI2) of the S-layer on cells (Fig. 4*C*). Although not resolved in our map, an averaged density profile perpendicular to the OM shows a small peak that may correspond to HPI1 (Fig. 4*C*) where the S-layer is attached to the OM. Also, both large gaps between hexamers (~76 Å) and small gaps at the hexameric pore (~33 Å) were observed in our subtomogram average, consistent with our cryo-EM atomic model.

Cryotomograms also confirmed that HPI2 is positioned proximal to the cell membrane and, together with HPI1, plays a role in S-layer anchoring. Density for HPI1 was not resolved in our average, but a peak in the density profile proximal to the OM was observed that could be partially attributed to HPI. HPI3-HPI7 form the S-layer lattice (the canopy), with large pores exemplifying the lattice. Our in situ structure of the cellular S-layer also confirmed that HPI is the only protein forming the S-layer, as no density for additional proteins was observed in either our in vitro or in situ structures.

### Updated Model of the *D. radiodurans* Cell Surface.

**Fig. 5.**
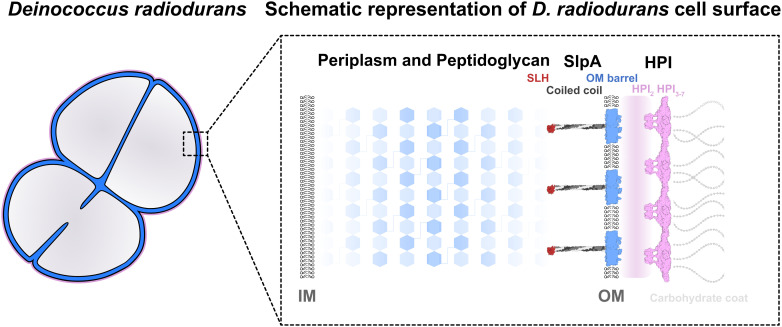
Model of the *D. radiodurans* cell envelope. Schematic model of the *D. radiodurans* cell envelope shows how HPI protein forms the S-layer on cells, in line with previous reports (8, 9, 11–13, 18–22). HPI3-7 form the S-layer lattice farthest away from the cell, while HPI1-2 are closer to and probably tethered to the OM via lipidation of an N-terminal cysteine residue in HPI1, whose exact spatial location within the cell envelope was not resolved. An abundant protein called SlpA protein is buried in the OM, connecting it to the PG layer via long coiled coils and an N-terminal SLH domain, as shown previously (37, 39). Our results place previous studies into context, and our updated model of the *D. radiodurans* cell envelope will serve as a framework for understanding the cell surface organization of phylogenetically deep-branching bacteria.

Using our atomic and cellular data, we report an updated model of the *D. radiodurans* cell surface (Fig. 5). Our data are consistent with those of previous models of the *D. radiodurans* envelope produced primarily by negative-stain EM (8, 9, 11–13, 20–22). The analysis of cryo-ET data from FIB-milled cells further shows that HPI is the sole protein that forms the S-layer. We show that no densities are present for the highly abundant OM SlpA protein (37), which belongs to the OmpM superfamily of PG-OM tethering proteins (38), consistent with previous reports (8, 9, 11–13, 20–22). Moreover, our data from FIB-milled cells indicate that the S-layer is positioned ~18 nm away from the OM, which is in close agreement with recent cryo-EM studies (10, 37). Our atomic structure of the S-layer and the fit of this structure to the subtomogram averaging map of the cellular S-layer show that this lattice coats *D. radiodurans* cells as a highly interconnected sheet with exceptional stability (Fig. 5). Thus, this updated model of the *D. radiodurans* cell envelope allows us to put previous EM studies into the context of atomic structures.

## Discussion

The cell envelope of *D. radiodurans* has been studied for over five decades because of its hyperstability and uniqueness compared to other bacteria (17, 22, 37, 39). Numerous studies have employed a variety of approaches to obtain structural and molecular information on the *D. radiodurans* S-layer, its composition (13, 20), symmetry (9, 17, 22), and overall arrangement (11, 12). Here, we build on this knowledge by applying the latest technological advancements in structural biology (40, 41) to obtain an atomic model of the native arrangement of the HPI S-layer.

Our structure places several previous studies into context and confirms that the *D. radiodurans* S-layer is composed exclusively of the HPI protein. A recent model of the *D. radiodurans* S-layer proposes that it contains another protein called SlpA (42–44). However, this proposal is incompatible with our data and also with previous genetic (39), biochemical (45), and EM studies (8, 9, 11–13, 20–22) that have shown that SlpA is a PG-OM tether (39, 45) and that the S-layer consists exclusively of the HPI protein (8, 9, 11–13, 20–22). Our data support these previous studies in that SlpA stabilizes the cell envelope by connecting the OM to the PG (37). The proposal that HPI is the only protein in the S-layer is supported by our atomic structure (Figs. 1 and 2) and by our in situ cryo-ET and subtomogram averaging of native cells (Fig. 4), which showed no density corresponding to SlpA in the S-layer.

The HPI protein consists of seven consecutive, evolutionarily divergent Ig-like domains. This organization has been previously observed in Gram-positive bacterial (23, 24) and archaeal (26) SLPs, suggesting a unique ability of Ig-like domains to support diverse functions at the cell surface. It is tempting to speculate that these Ig-like domains may have facilitated the evolution of more complex surface recognition modules (46), such as the eukaryotic immune system. Indeed, glycosylation patterns on archaeal SLPs are known to help organisms recognize “self” from “nonself” (47). Primordial S-layers composed of such Ig domain-containing SLPs may have played an important role in cellular recognition, which in turn may have supported the evolution of the modern eukaryotic cell, as suggested previously (46). In the case of *D. radiodurans*, it is possible that the S-layer also plays a role in cellular recognition. Unraveling the functional role of this and other S-layers is an outstanding question in this field that should be urgently addressed in future studies.

The structure of the *D. radiodurans* S-layer also reveals how this sheet-like assembly displays hyperstability. The domain-swapped arrangement of the hexagonal HPI lattice, in which a single polypeptide chain is shared with adjoining hexamers, leads to a highly interconnected structure that would require multiple biochemical steps for complete disassembly into monomers. Additionally, the lattice is stabilized by many salt bridges that provide exceptional stability even in the presence of strong denaturing detergents such as SDS (8, 13). How the S-layer is bound to the OM is unclear because residues 19 to 132 of the mature HPI protein were not resolved in our cryo-EM map. However, our bioinformatic analysis suggests that an invariant cysteine representing the first residue of the mature HPI protein may be lipidated, consistent with previous reports finding that S-layer preparations stain positive for lipids (9).

This hyperstable S-layer structure of *D. radiodurans* could be exploited for applications in synthetic biology, such as surface presentation of molecules in high-copy number on cells and in vitro, as has been described for other S-layers (30, 48). This structure also provides important insights into the organization of the enigmatic cell envelope of a phylogenetically deep-branching bacterium and will help illuminate our understanding of the evolution of Gram-negative and Gram-positive bacteria by providing the basis for future studies on the arrangement and evolution of prokaryotic cell surfaces.

## Materials and Methods

### Purification of HPI Protein.

Wild-type HPI protein was purified from *D. radiodurans* bacteria by adapting a previously described protocol (13). Six liters of modified tryptone-glucose-yeast (TGY extract) medium was inoculated 1:50 with a late-log phase culture of *D. radiodurans*, and cells were grown aerobically at 30 °C. Late-log phase cells were harvested by centrifugation [5,000 relative centrifugal force (rcf), 4 °C, 30 min] and frozen and stored at −80 °C until further experimentation. The cell pellet from a 1-L culture was carefully resuspended in 80 mL milliQ water, supplemented with 1× cOmplete protease inhibitor cocktail (Roche). The HPI layer was released from the cell surface by adding dropwise a 10% (w/v) SDS stock solution to the cell suspension up to a final concentration of 2% (w/v) SDS. The cell suspension was incubated for 2 h at room temperature on a rotatory wheel, and solubilized protein was separated from HPI and cell debris by centrifugation (8,000 rcf, 25 °C 20 min). The remaining pink pellet was carefully resuspended in 100 mL 2% (w/v) SDS and HPI was separated from stripped cells by centrifugation (3,000 rcf, 25 °C 20 min). The remaining supernatant containing HPI was carefully removed from the cellular pellet, and HPI sheets were subsequently centrifuged (35,000 rcf 25 °C 20 min), forming a small opaque pellet at the bottom of the centrifugation tube. The pellet was washed four times by resuspending the pellet in a buffer containing 50 mM 4-(2-hydroxyethyl)-1-piperazineethanesulfonic acid (HEPES)/NaOH pH = 7.5, 150 mM NaCl, 5 mM MgCl_2_, and 1 mM CaCl_2_ followed by centrifugation at 16,000 rcf 4 °C, 15 min. The final pellet was resuspended in the same buffer and the protein concentration was measured by ultraviolet absorption at 280 nm as 4.7 mg/mL. This protein solution was then used for cryo-EM experiments.

### Cryo-EM Sample Preparation.

For cryo-EM grid preparation of purified protein, 2.5 µL of the specimen was applied to a freshly glow-discharged Quantifoil R2/2 Cu/Rh 200 mesh grid, adsorbed for 60 s, blotted for 4 to 5 s, and plunge-frozen into liquid ethane in a Vitrobot Mark IV (ThermoFisher), while the blotting chamber was maintained at 100% humidity at 10 °C. Grids for cryo-FIB milling of *D. radiodurans* cells were prepared as described previously (10). Briefly, *D. radiodurans* strain BAA-816 (obtained from the American Type Culture Collection) was grown aerobically in TGY liquid medium (49). Cells were grown for 24 h at 30°C prior to harvesting and staining with FM4-64 fluorescent membrane dye (Invitrogen). Four microliter of cells was loaded on Finder grids (Electron Microscopy Sciences) and plunge-frozen in a liquid ethane–propane mixture kept at liquid nitrogen temperatures using a Vitrobot Mark IV (Thermo Fisher Scientific). Grids were clipped and stored under liquid nitrogen.

### Cryo-EM and Cryo-ET Data Collection.

#### Cryo-EM single-particle data.

Single-particle cryo-EM data were collected as described previously (26, 31, 37) on a Titan Krios G3 microscope (ThermoFisher) operating at 300 kV fitted with a Quantum energy filter (slit width 20 eV) and a K3 direct electron detector (Gatan) with a sampling pixel size of 0.546 Å running in counting superresolution mode. For the HPI S-layer sheets sample used for the hexameric lattice structure determination, a total of 1,002 movies were collected with a dose rate of 4.6 e^−^/pixel/s on the camera level. The sample was subjected to 3.43 s of exposure, during which a total dose of 53.3 e^−^/Å^2^ was applied, and 40 frames were recorded per movie (*SI Appendix*, Table S1).

#### Cryo-ET data.

Cryo-FIB milling of the specimen was performed as described previously (10) using a Zeiss Crossbeam 550 FIB-SEM microscope and generated 200-nm thick lamellae. Subsequently, the EM grids were transferred to a Titan Krios transmission electron microscope operating at 300 kV, equipped with a Falcon 3 direct electron detector (Thermo Fisher Scientific) for cryo-ET data collection. Tilt series images were acquired bidirectionally using the SerialEM software (50) at 18,000× or 22,500× magnification (pixel sizes 4.6 Å or 3.7 Å, respectively) with a defocus of −4 μm, ±60° oscillation, 1° increments with a total final dose of 100 e^−^/Å^2^.

### Cryo-EM Single-Particle Analysis.

#### HPI structure from two-dimensional sheets.

Cryo-EM data processing was performed as described previously for two-dimensional S-layer sheets (26). Movies were clustered into optics groups based on the XML metadata of the data collection software EPU (Thermo Fisher Scientific) using a k-means algorithm implemented in EPU_group_AFIS (https://github.com/DustinMorado/EPU_group_AFIS). Imported movies were motion-corrected, dose-weighted, and Fourier-cropped (2×) with MotionCor2 (51) implemented in RELION3.1 (52). Contrast transfer functions of the resulting motion-corrected micrographs were estimated using CTFFIND4 (53). Initially, side views of S-layer sheets were first manually picked along the edge of the lattice using the helical picking tab in RELION while setting the helical rise to 40 Å. Top and tilted views were manually picked at the central hexameric axis. Manually picked particles were extracted in 4× downsampled 100 × 100 boxes and classified using reference-free 2D classification inside RELION3.1. Class averages centered at a hexameric axis were used to automatically pick particles inside RELION3.1. Automatically picked particles were extracted in 4× downsampled 100 × 100 pixel^2^ boxes and classified using reference-free 2D classification. Particle coordinates belonging to class averages centered at the hexameric axis were used to train TOPAZ (54) in 5× downsampled micrographs with the neural network architecture conv127. For the final reconstruction, particles were picked using TOPAZ and the previously trained neural network above. Additionally, top, bottom, and side views were picked using the reference-based autopicker inside RELION3.1, which TOPAZ did not readily identify. Particles were extracted in 4× downsampled 100 × 100 pixel^2^ boxes and classified using reference-free 2D classification inside RELION3.1. Particles belonging to class averages centered at the hexameric axis were combined, and particles within 30 Å were removed to prevent duplication after alignment. All resulting particles were then reextracted in 4× downsampled 100 × 100 pixel^2^ boxes. All side views and a subset of top and bottom views were used for initial model generation in RELION-3.1. The scaled and low-pass filtered output was then used as a starting model for 3D auto refinement in a 512 × 512 pixel^2^ box. Per-particle defocus, anisotropy magnification, and higher-order aberrations (55) were refined inside RELION3.1, followed by another round of focused 3D autorefinement and Bayesian particle polishing (55). The final map was obtained from 55,345 particles and postprocessed using a soft mask focused on the central hexamer, including the dimeric bridge, yielding a global resolution of 2.5 Å according to the gold standard Fourier shell correlation criterion of 0.143 (56). The two-dimensional sheet-like arrangement led to anisotropy in resolution, with lower resolution perpendicular to the plane as estimated by directional Fourier Shell Correlations (FSCs) (57), observed previously by several studies on two-dimensional sheets (58, 59). See also *SI Appendix*, Fig. S1 and Table S1 for further details.

### Cryo-ET Data Analysis.

Tilt series alignment using patch tracking and tomogram generation was performed using IMOD (60). Subtomogram averaging was performed using custom scripts written in MATLAB (MathWorks), described previously (35, 61, 62). For the preliminary assignment of angles and initial structure determination, we adopted established methods (32). This workflow allowed us to produce lattice maps from cells. Initial subtomogram averaging maps and output angular angle assignments were then used to refine the subtomogram averages further, resulting in the final maps of the HPI hexamer from cells. Figure panels containing cryo-EM or cryo-ET images were prepared using IMOD and Fiji (63). Lattice maps of S-layers for visual inspection were plotted inside University of California at San Francisco Chimera (64) with the *PlaceObject* Plugin (65). Movie S1 was prepared with UCSF ChimeraX.

### Model Building and Refinement.

#### HPI hexameric structure from sheets.

For model building, the original 512 × 512 × 512 voxel box was cropped into a 400 × 400 × 400 voxel box and the protein backbone of HPI was manually traced as a polyalanine model through a single HPI subunit using Coot (66). Side chains were assigned at clearly identifiable positions which allowed deduction of the protein sequence register. The atomic model was then placed into the hexameric map as six copies and subjected to several rounds of refinement using refmac5 (67) inside the Collaborative Computational Project for electron cryo-microscopy (CCP-EM) software suite (68) and PHENIX (69), followed by manually rebuilding in Coot (66). HPI5 formed an extended bridge at the edge of the hexamer, and for that part of the map, special considerations were used for model building. The map in this region was locally sharpened using *servalcat* (70) and this map was additionally rotated using a local twofold axis to confirm our model building. Model validation was performed in PHENIX and CCP-EM, and data visualization was performed in Chimera, ChimeraX, and PyMOL (71). To analyze lattice interfaces, multiple copies of the hexameric structure were placed in the cryo-EM map prepared with a larger box size.

### Bioinformatic Analysis.

All sequence similarity searches were performed in the MPI Bioinformatics Toolkit (72) using BLAST (73) and HHpred (74). BLAST searches were performed against the nr_bac database, a version of the NCBI nonredundant protein sequence database (nr) filtered for bacterial sequences, using default settings to identify homologs of HPI in bacteria. The searches were seeded with the protein sequence of *D. radiodurans* HPI (UniProt ID P56867). The domain organization of several obtained matches and many experimentally characterized SLPs (*SI Appendix*, Table S2) was analyzed using HHpred searches in default settings over the PDB70 and ECOD70 databases (versions of the Protein Data Bank and ECOD databases filtered for a maximum pairwise identity of 70%) and using structural models built with AlphaFold v2.2.0 using the “monomer_ptm” model (27). Signal peptides were predicted using SignalP 6.0 (75).

## Supplementary Material

Appendix 01 (PDF)

Movie S1. **Atomic structure of the *D. radiodurans* S-layer.** The cryo-EM map and atomic structure of the *D. radiodurans* S-layer show how the immunoglobulin-like domains of HPI form the lattice. Different views of the S-layer are shown with text annotations.

## Data Availability

All study data are included in the article and/or supporting information, reasonable requests for data will be fulfilled by Tanmay A.M. Bharat. The HPI cryo-EM map has been deposited in the Electron Microscopy Data Bank (EMDB) with the accession code EMD-16694 (76) and the corresponding refined atomic model has been deposited in the Protein Data Bank (PDB) with the accession code 8CKA (77). For further details see *SI Appendix*, Table S1.
