# Supplementary material for: Interdigitated immunoglobulin arrays form the hyperstable surface layer of the extremophilic bacterium Deinococcus radiodurans
Source: Proc Natl Acad Sci U S A. 2023 Apr 12;120(16):e2215808120. doi: 10.1073/pnas.2215808120 (PMC10120038; doi:10.1073/pnas.2215808120)
Supplement: Supplementary file 1 — Appendix 01 (PDF) [file pnas.2215808120.sapp.pdf]

PNAS
www.pnas.org

**Supplementary Information for**

**Title**

Interdigitated immunoglobulin arrays form the hyperstable surface layer of the extremophilic bacterium *Deinococcus radiodurans*

**Authors**

Andriko von Kügelgen[1,2], Sofie van Dorst[2], Keitaro Yamashita[1], Danielle L. Sexton[4], Elitza I. Tocheva[4], Garib Murshudov[1], Vikram Alva[3] and Tanmay A. M. Bharat[1,2,*]

**Affiliations**

1 – Structural Studies Division, MRC Laboratory of Molecular Biology, Francis Crick Avenue, Cambridge CB2 0QH, United Kingdom

2 – Sir William Dunn School of Pathology, University of Oxford, Oxford OX1 3RE, United Kingdom

3 – Department of Protein Evolution, Max Planck Institute for Biology Tübingen, Max-Planck-Ring 5, Tübingen 72076, Germany

4 – Department of Microbiology and Immunology, University of British Columbia, Vancouver, Canada V6T 1Z3

* **Correspondence to** Tanmay A.M. Bharat

**Email:** tbharat@mrc-lmb.cam.ac.uk

**This PDF file includes:**

Figures S1 to S6

Tables S1 to S2

Legend for Movie S1

SI References

**Other supplementary materials for this manuscript include the following:**

Movie S1

## Supplementary Figures

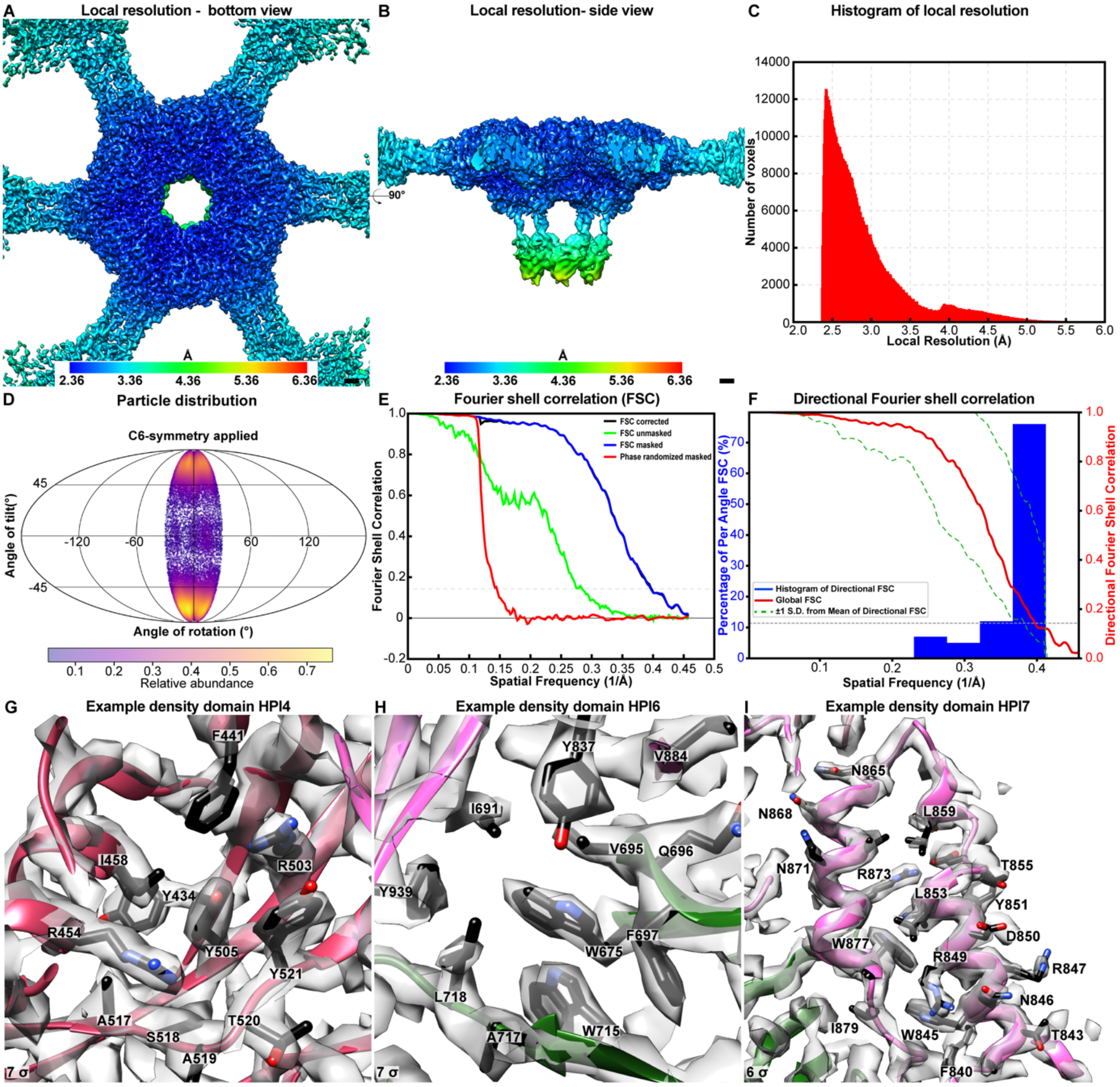


**Figure S1. Cryo-EM structure of the *D. radiodurans* S-layer.**

(A-B) Local resolution of the cryo-EM map estimated in RELION, plotted into the density, shown in two orthogonal orientations. The resolution of HPI2 is slightly lower. (C) Histogram of local resolutions in voxels of the cryo-EM map. (D) Angular distribution of the particles in the data set, shown on a relative scale (purple denotes low and yellow denotes high). (E) Fourier Shell Correlation (FSC) estimation of the resolution of the map. (F) 3D FSC between two random halves of the data (1). (G-I) Examples of the cryo-EM density and the fit of the model into the map. Contour levels are displayed on the bottom left. Scale bars: A-B) 10 Å.

```
                      lipobox with a conserved cysteine
D. radiodurans    MKKNIALMALTGILTLASCGQNGTGTTPTADACATAN---------------------------------------------------------------
D. phoenicis      MKKNMALMALTGILTLASCGGNTPTDNSGTQVVD------------------------------------------------------------------
D. wulumuqiensis  MKKNIALMALTGVLTLASCGQTGNTPTADTTA------------PTVSLSVNNANLPSAGGSVVLSGTVNEASTVVVKNNAGATVCTVEVAASGTFTCPA
D. murrayi        MNKNIALLALTGVLTLASCGTQPTPTPTPTPTPTPTPTPTPTPT--------------------------------------------------------

                                                                D1<----------------------------------------------------
D. radiodurans    ----------------------------------------------TCSVTVNIS-GVSSADFDVTM-DGKT---TS-MTLSNGQKLPVAKTGTVTLTPK
D. phoenicis      -----------------------------------------------RTLTVNLTGGVTSAPITVTDTNGVT---SS-FTVNNGQVLTL-KNKKYTVTGG
D. wulumuqiensis  TTIAGNTSTTSTSTSYTATATDAAKNVGTSSVVTVNVAGVSNPAPTTAVLTIDLA-GVSSAPITIKDANGNVVQGYDNVTVNDNATITV-ARGVYTVTAG
D. murrayi        ----------------------------------------------------------------------------------------------------

                  ----------------------------->D1                 D2<------------------------------------------------
D. radiodurans    AKDGYTTP-AAQSTTISSTNLTPSVNFAYTTVPSTGNGNGNGGTTPTQPFTLNITSPTNGAAATTGTPIRVVFTSSVALSSATCKIGN----SAAVNAQV
D. phoenicis      TVNGYTSP-NSQTADLTN--GNGSVTLNYTASNPGTN----PNPNPNPSAVLNITSPTNGASVTMNQAIQVTFTTSAALTGVTCTIAD----GTAVNASS
D. wulumuqiensis  NVSGFNGPTTNFRVDLS--GGNQTVTLNYTQA---------GTTTPTPVGSINILTPAVGTSVTGGSTVRVTF-DKANVNEVQCMVGG----AAAVTAQV
D. murrayi        -----------------------------------------PTPTPTPTNSISITSPTQGGNATVGQAIQVNYTTSAPLTNVTCTIAAGGNTSSPATANN

                  ----------------------------------------->D2   D3<--------------------------------------------------
D. radiodurans    SSTGGYCD-VTPTTAGGGLITVTGT-ANGQTVSSTVTVDVKAPVVD-NRYGTVTPAGDQELTLTNEGIVKDADNGWRRLGQGVSTPSDPNGNVDIYVKGT
D. phoenicis      STSGGFCT-VTPTVAGSNVITVSGKDANGNTVSSSVAVDAKAPATPATSNISFDPT--QELTLTNEGIVIDAVNGWRRIGQGVSTPGNPDTNVDVYVKGT
D. wulumuqiensis  DSTSGYCDVVVPNSTGNVVITVMGKGVNGQTVTATRNISVTQAAVS---YGVVTPAGDQELTLTSEGIVRDADSGWRRLGQGVSTPSDPNLNLDIYIKGT
D. murrayi        STSGGFCT-VTPTAAGSNIITVTGTDAQGRTVTQQVVVNAVAAPTPATSNIVFDPA--QELTLTSEGIVREAANGWRRIGQGVSTSTSPETNVDVYVKGT

                  ----------------------------------------------------------------------------------->D3  D4<---------
D. radiodurans    VNFSV-NAAAGSKVEVFLARTTGSDVPTNDDVQAGDVLRSVASTSGTETFSLDSRRLAEFDGVRKWIVVRINGTQVTYQPVIADNKGPQQPDPELNGVQN
D. phoenicis      VNVSY-TAAAGSKVEIILARTTGSDVPTNDDIQAGDVLRAVASANGPVTAQLDTRRLGEFEAVREWLVFRVNGTAVSFQPVIADNKGPQQADPEFNGVSN
D. wulumuqiensis  VNFSV-NAPAGQKVEVFLARTTGSDVPTNDDIQAGDVLRSVASTSGTETFSLDSRRLAEFDGVRKWIVVRINGTQVTYQPVIADNKGPQQPDPELNGVQN
D. murrayi        VNVSYRGAAAGQKVEIILARTTGSDAPSNDQIQAGDVLRSVVSTGADVTAVYDSRRLAEFEGVRQWLVFRVNGTVVSFQPVITDNRGPQQPDPEFNGVNN

                  ----------------------------------------------------------------------------------------------------
D. radiodurans    AYSNILNNYNNSGLTYVRGDVNVFTGNPSLQDREFGQAPLGSSFVQRRPSGFESIRYYLVPETAFGNKALQESDEMLRAKAIKSVATVVSAPVLEPGTVK
D. phoenicis      GYSALRRNYNGTNINFARGDINLFTSNPSLQDREYGQAPYGASFLQRRPAGFESIRYYLVPETAFNDNGLQDSDEMRRAQAIKSVATVKSAAILEPGSDR
D. wulumuqiensis  AYSNILNNYNNSGLTYVRGDVNVFTSNPSLQDREFGQAPVGSSFVQRRPAGFESIRYYLVPESAFNNKALQESDEMLRAKAVKSVATVVSAPVLEPGTVK
D. murrayi        TYSNLLRNVDNTGINWARGDINVFTSNPSLQDREFGQAPVGASFAQRRPSGFESIRYYLVPETAFDNKGLADSDEMRRAEAVKSAATARSAPILEPG-DR

                  ------------------------------------------------------>D4 D5<---------------------------------------
D. radiodurans    ATSFSRVIGSGATSTV--TPKA--QDNVTYRVYAISRDQLGNETASATYELVRFDNVGPTITGSVIRDTSDLPFASQEPERCLSDIATITLGGITDNAGG
D. phoenicis      STAFSSMIGSGSQTTVGTAQRA--LDNVNYRIYTIVRDQLGNETASASYETIRFDNVGPVITGSALRDTSALPFPSLEPERCLSDIATVNLGGATDNEGG
D. wulumuqiensis  ATSFSRVIGSGATSTV--APKA--LDNVTYRVYAISRDQVGNETASATYELVRFDNVGPTITGSVIRDTSDLPFPSQEPERCLSDIATITLGGIADNVGG
D. murrayi        SAAFSSLFGSTGATATGATRTAGVQDGVTYRIYAISRDQLGNETASASYETIRFDNVGPSFTGSVLRDASDLPFPSVEPERCLSDYATVNLGSATDNPGG

                  ---------------------------------------------------------->D5  D6<----------------------------------
D. radiodurans    VGLNPGQGLTFTLGGRQIQAGQ-FDTNQLADGEYTIGFNSLTDALGNPVVSAPTNAKVYIDNTDPTVNFNRAVMQGTFASGERVSVESDASDGGCGVYET
D. phoenicis      IGV-IGQ-PTFNIGGINLTNGEAFDTNRLADGQYKIDYGTLTDALGNPATGA-VNNTVFIDNTDPTVNFNRPALQSVVNSGERVSVESSASDGGCGVYEA
D. wulumuqiensis  VGLNPGQGLTFTLGGRQIQAGQ-FDTNQLADGEYTIGFNSLTDALGNPVVTAPTNAKVYIDNTDPTVNFNRAVLQSTYASGERVSVESDSSDGGCGVYET
D. murrayi        IGI--GSQPTFTIGGITLNGQTTFDTNRLADGEYTINTSNFTDRLGNPPVNAPQNATVFIDNTDPTGNFNRPVVGGVFDSGERVSAETDFSDGGCGVHEI

                  ---------------------------------------------------------------------------------------->D6
D. radiodurans    RLFWDTD------NGVVDDATTTPAIGHPVQFARQRVTDGA-KADSLNAGWNALQLPNGAGAVYLRALVVDRAGNATISTTPIVVNAKI-----------
D. phoenicis      RLFWDTNISTNGAPGAIDDG-TTDTIGHPVQFARRTADAGS-SSMSLNQGWNALQVPGEGGIVQLRAMVTDRAGNATIVSTPIVIVPKTGKIDNGAGVIS
D. wulumuqiensis  RLFWDTA------NGVVDDATTTPAIGHPVQFARQRVTDGA-KADSLNAGWNALQLPNGAGAVYLRALVVDRAGNATISTTSIVVNAKI-----------
D. murrayi        RLFWDTNI-----TNATDDETTTPGIGHPVQFARSAQSTGRMTSMSFNGGWNALQLPNLRSEVRLRALAIDRAGNATLVSTPIVVNAKG--------LGS

                        D7<-------------------------------------------------------------------------------------------
D. radiodurans    ------TNQARPLLGGFDAFKRNASAQFMSNSNAIS--GVNGTAVTPNTTANSALDNILSLDSVGTLTTNAY--LPRGATETAITEKIRNVGAYGRFDAT
D. phoenicis      TV---TINNARPTLGLSDGYLRNTSAVFGNTSNTIAGLGANGTVLVVNTTGNSTLDNNLTLDATGTFTTNGFPNQPLPSPTPNLVEKINAVMAYGRFDAA
D. wulumuqiensis  ------TNQARPLLGGFDAFKRNASAQFVGDDNVIA--GVNGTAATPNVTGNSALDNILSLDSVGTLTTNAY--LPRGATETAITEKIRNVGAYGRFDAT
D. murrayi        SAQPGGVNTNRIILGNSDGYRRNQAINLQPNTNLIAAFGANGTTLIPNTTGNSLLDNNLSLDSTTTRTVN--------STGNTGSENIREVSDYGRFDAA

                  ----------------------------------------------------------------------------------------------------
D. radiodurans    QWNRIRDYQLNTDPTLRSAYVNA--GNLANQRGNNWRIRTPWVELGSSDTANTQQKFDFNSDLLNDFYFGRTFGNND-----------------NVNLFS
D. phoenicis      QWAAIRDYQRAGDPTLTSPNATN--SGLANQRVTNWSIRSPWVALGDSATADNKQQYTFTSNLLNDFYNNRGFPNTG-----------NDDAVLSTDAVS
D. wulumuqiensis  QWNLIRDYQLNTDPTLRSAYVNA--GNLANQRGNNWRIRTPWVELGSSDTANTQQKFDFNSDLLNDFYYGRTFGNNH-----------------SVNLFS
D. murrayi        LWNTIRNYQLRDDPTLNSGNRTQDIGQLLSQRQRNWNVRTPWIYITDSTTPDNKQLYTATSDLLNDFYWGRTFRNPAANTNMVGSTVINDVSVFNTNRFT

                  ----------------------->D7
D. radiodurans    YDQFNGIVSGTAGAYSFYGETVQK------
D. phoenicis      YDQFNGIVTDTAGSYSFFGEEADTTATP--
D. wulumuqiensis  YDQFNGIVSDTAGAYSFYGETVRK------
D. murrayi        YDQFNKLVADTAGAYSFLGERVGNVDGTAR
```

**Figure S2. Multiple sequence alignment of *Deinococcal* HPI SLPs.**

The four-residue-long lipobox motif comprising an invariant cysteine residue is colored pink, whereas positively- and negatively-charged residues observed to form salt bridges in our structure are colored blue and red, respectively. The domain boundaries of the seven Ig-like domains are indicated, and cysteine residues in close proximity in the model are colored green. Accession details for the shown protein sequences are provided in Table S2.

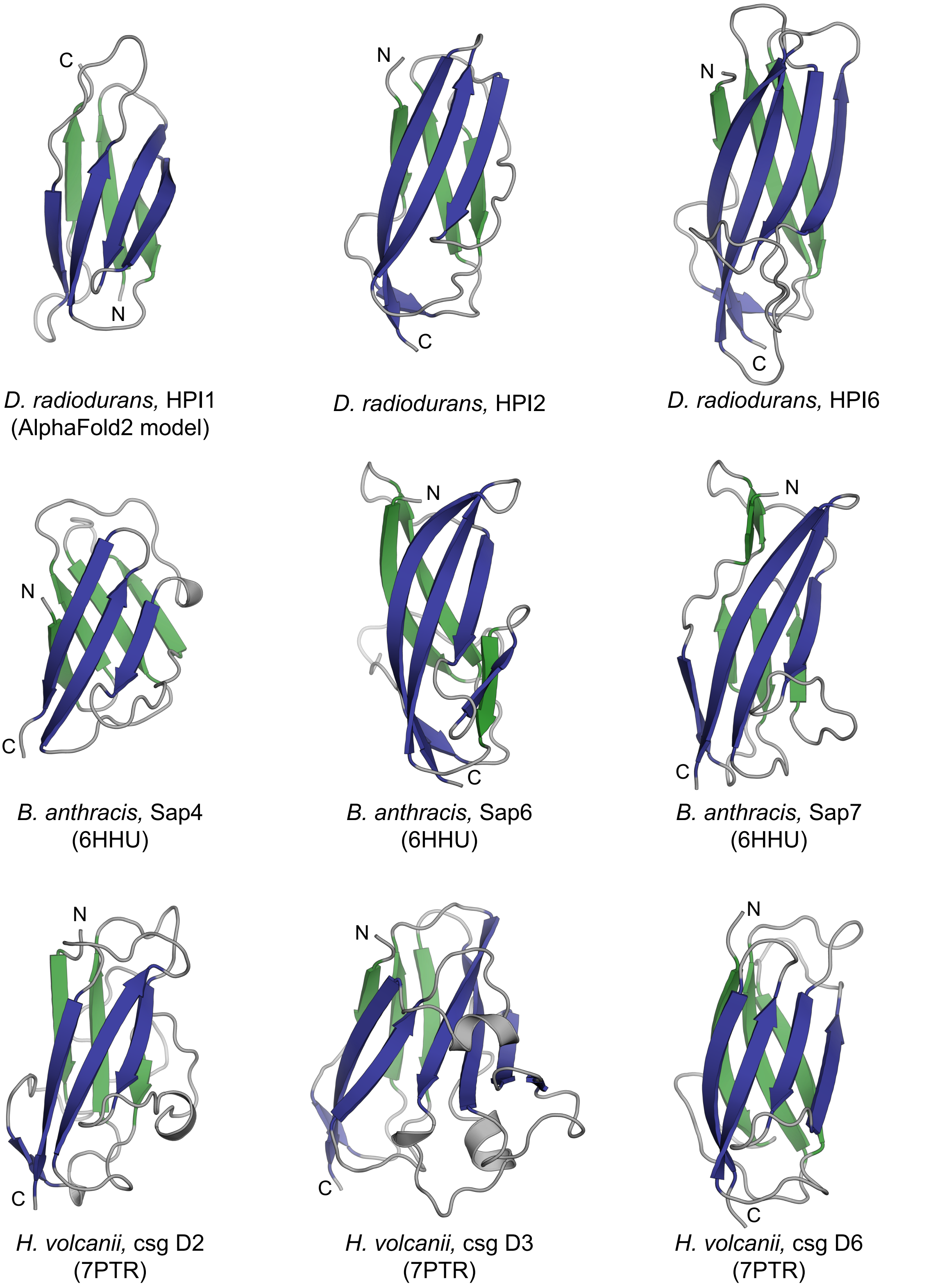


**Figure S3. Gallery of representative Ig-like domains from bacterial and archaeal SLPs.** The structure of *D. radiodurans* HPI1 was predicted using AlphaFold2; the PDB accessions of other shown structures are provided within the rounded brackets. Although quite divergent in

their sequences, the Ig-like domains of many prokaryotic SLPs exhibit topologically similar folds.

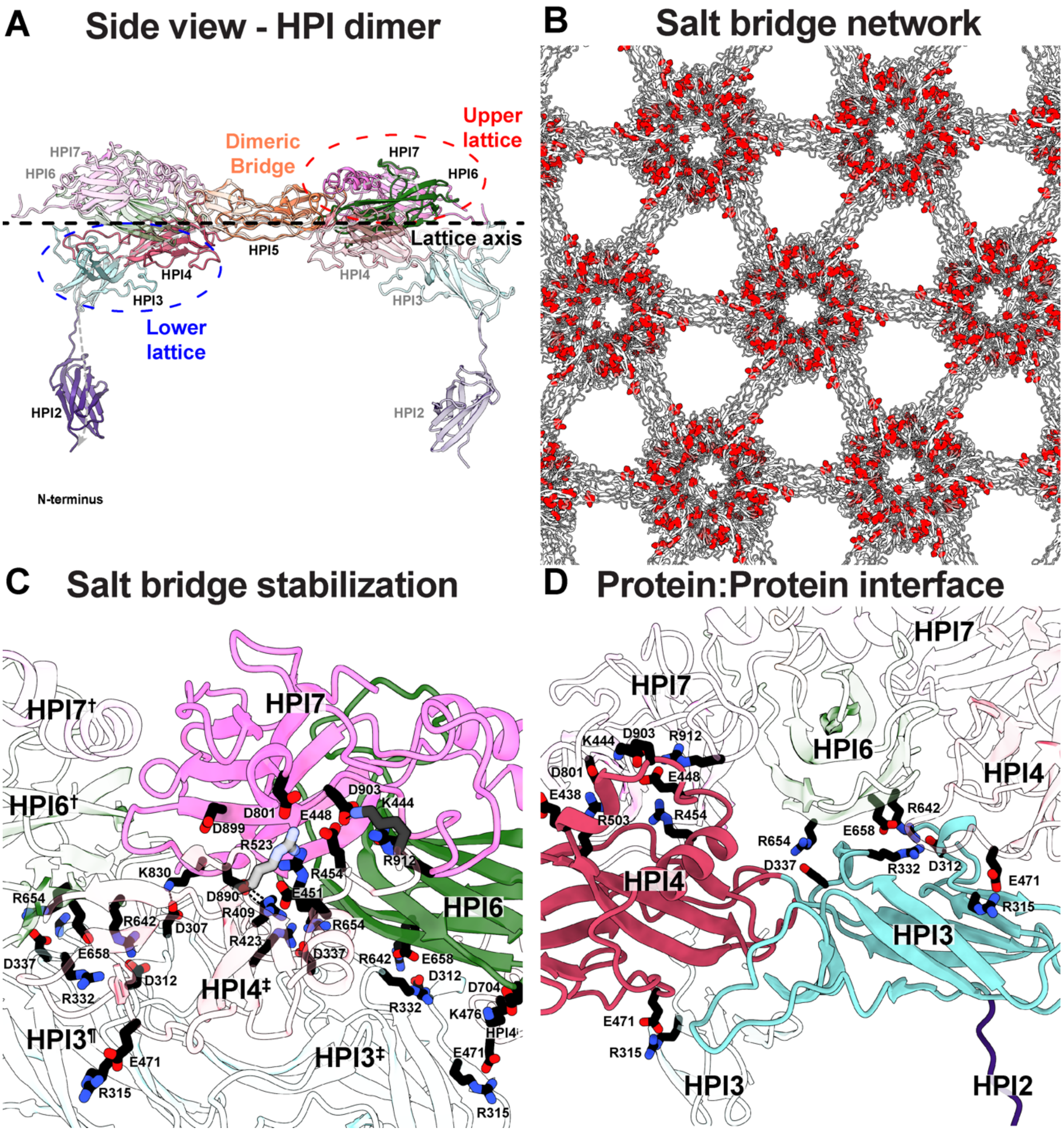


**Figure S4. The HPI S-layer lattice arrangement.**

(A) Side view of the HPI lattice dimer shows how the S-layer is arranged in multiple layers. HPI6-7 form the upper layer (red dashed ellipse), connected to a lower lattice made up of HPI3-4 (blue dashed ellipse) through bridging dimeric HPI5 domains (orange). HPI2 forms the lowest layer resolved in the map proximal to the cell. (B) The HPI lattice is shown from the top (from outside the cell), with HPI protein residues forming salt bridges colored in red. This extensive salt bridging network stabilizes the lattice. (C-D) The lattice is stabilized by multiple salt bridges, shown in zoomed-out views of the Figure 2D-E.

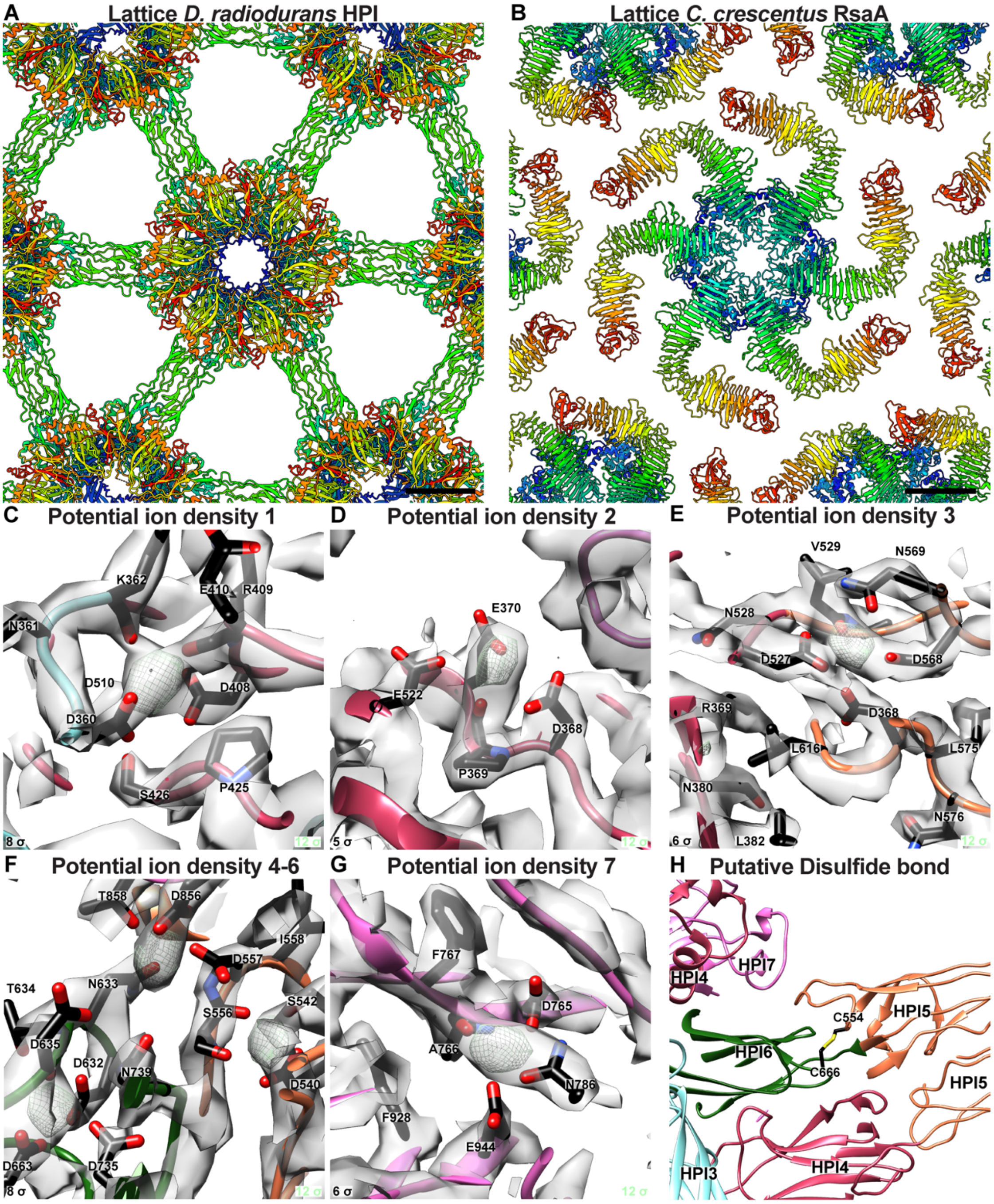


**Figure S5. Gaps and putative ion densities observed in the lattice.**

(A) Top view of the HPI lattice shows several large gaps (pores) in the S-layer (Scale bar 50 Å). Each HPI monomer is colored as rainbow from the N- to the C-terminus. (B) For comparison, a top view of the previously reported outer S-layer lattice from the diderm *C. crescentus* bacterium, where smaller pores are observed. Each SLP monomer (RsaA protein) is colored as rainbow from the N- to the C-terminus (Scale bar 50 Å). (C-G) In several locations, negatively charged residues were bound to unexplained densities, which could be

putative cations. Contour levels of cryo-EM density (grey, isosurface) and Fo-Fc map calculate by *servalcat* (2) is displayed on the bottom left and right, respectively. (H) Two cysteine residues (C554 and C666) are placed in close proximity in our model, which may further interconnect the lattice with a disulfide bond.

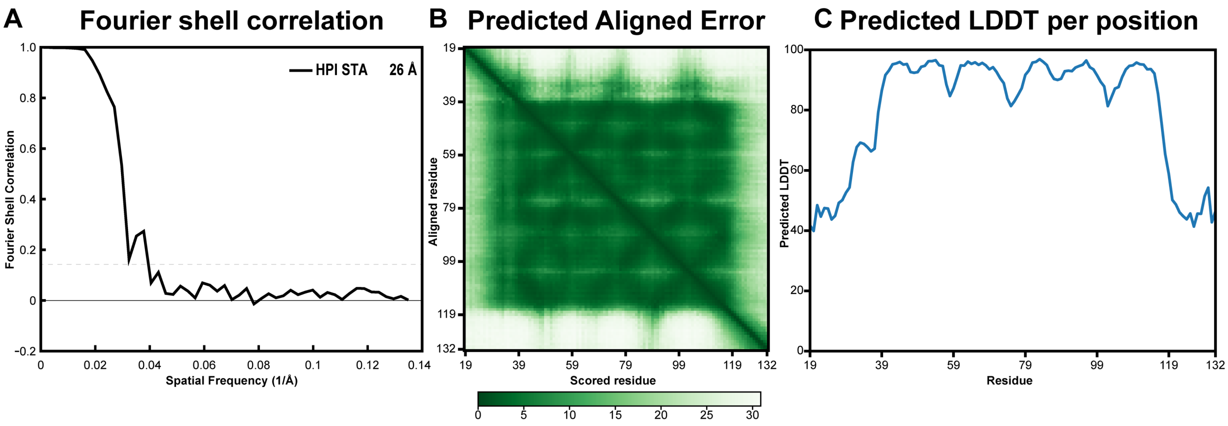


**Figure S6. Resolution estimation and structure validation.**

(A) Fourier Shell Correlation (FSC) of independently aligned and averaged halves of the *in-situ* HPI data estimates resolution of the map at 26 Å. (B) The Predicted Aligned Error (PAE) and (C) per-residue confidence (pLDDT) plots for HPI1 AlphaFold2 prediction are shown. Low PAE and high pLDDT values are indicators for high accuracy of the model yielded by AlphaFold2 (3).

**Supplementary Tables**

**Table S1: Cryo-EM data collection, refinement and validation statistics**

| | #Drad_HPI<br>(EMD-16694)<br>(PDB 8CKA) |
|---|---|
| **Data collection and processing** | |
| Microscope | Titan Krios G3 |
| Magnification | 81,000 |
| Voltage (kV) | 300 |
| Total Electron exposure ($e^{-}/Å^{2}$) | 53.245 |
| Detector | K3 (Gatan) |
| Slit width (eV) | 20 |
| Defocus range (μm) | -1 to -2.5 |
| Acquisition Mode | Super-resolution |
| Pixel size (Å) | 0.546 |
| AFIS[&] Mode | Yes |
| Micrographs collected | 1002 |
| Micrographs used | 1002 |
| **Data processing** | |
| Software reconstruction | RELION3.1 (4) |
| Software picking | TOPAZ (5) |
| Initial particle images (no.) | 882,866 |
| Final particle images (no.) | 55,345 |
| Rescaled Box-size Class2D (px) | 100 x 100 |
| Rescaled Box-size Class3D (px) | 512 x 512 x 512 |
| Final refinement Box-size (px) | 512 x 512 x 512 |
| Final Box-size (px) | 400 x 400 x 400 |
| Pixel size final reconstruction (Å) | 1.092 |
| Symmetry imposed | C6 |
| Map resolution (Å) | 2.5 |
| FSC threshold | 0.143 |
| Map resolution range (Å) | 2.36-6.36 |
| Map sharpening *B* factor ($Å^{2}$) | -19.48 |
| 3D-FSC sphericity[#] | 0.897 |

| Model Refinement | |
|---|---|
| Initial model used (PDB code) | None |
| Software | Servalcat (2) |
| Model resolution (Å) | 2.9 |
| FSC threshold | 0.5 |
| Model composition | |
| Non-hydrogen atoms | 40,770 |
| Protein residues | 5,496 |
| *B* factors (Å$^2$) | |
| Protein | 76.53 |
| R.m.s. deviations | |
| Bond lengths (Å) | 0.007 |
| Bond angles (°) | 1.079 |
| Validation | |
| MolProbity score | 1.56 |
| Clashscore | 5.49 |
| Poor rotamers (%) | 0.27 |
| Cβ outliers (%) | 0.00 |
| CABLAM outliers (%) | 2.43 |
| Ramachandran plot | |
| Favored (%) | 96.15 |
| Allowed (%) | 3.85 |
| Disallowed (%) | 0.00 |
| Rama-Z (Z-score, RSMD) | |
| whole (N= 5448) | 0.36 (0.11) |
| helix (N= 318) | -0.02 (0.33) |
| sheet (N= 1848) | 1.20 (0.11) |
| loop (N= 3282) | -0.25 (0.10) |

[&] AFIS: Aberration Free Imaging Shift Mode.

[#] 3D-FSC sphericity as determined by the methods described in (1).

**Table S2: NCBI/UniProtKB IDs of prokaryotic SLPs containing Ig-like domain arrays.**

| **Organism (protein name)** | **NCBI/UniProtKB ID** |
|---|---|
| *Deinococcus radiodurans* (HPI) | P56867 |
| *Deinococcus phoenicis* (HPI) | WP_034356122.1 |
| *Deinococcus wulumuqiensis* (HPI) | WP_162865520.1 |
| *Deinococcus murrayi* (HPI) | WP_027460105.1 |
| *Deinobacterium chartae* (HPI-1) | WP_183984901.1 |
| *Deinobacterium chartae* (HPI-2) | WP_183984905.1 |
| *Deinococcus fonticola* (HPI) | WP_135229161.1 |
| *Thermus thermophilus* (HPI-like) | BDG20071.1 |
| *Bacillus anthracis* (Sap) | P49051 |
| *Bacillus anthracis* (EA1) | P94217 |
| *Geobacillus stearothermophilus* (SbsB) | CAA66724 |
| *Geobacillus stearothermophilus* (SbsC) | O68840 |
| *Paenibacillus alvei* (SpaA) | WP_005544766.1 |
| *Acetivibrio thermocellus* | O86999 |
| *Aeromonas salmonicida* | P35823 |
| *Haloferax volcanii* (csg) | P25062 |
| *Halobacterium salinarum* (csg) | P0DME1 |
| *Haloferax mediterranei* (csg-1) | WP_004057339.1 |
| *Haloferax mediterranei* (csg-2) | WP_004059902.1 |
| *Methanospirillum hungatei* | WP_011449226.1 |
| *Methanothermus fervidus* | P27373 |
| *Staphylothermus marinus* (Tetrabrachion) | Q54436 |
| *Aeropyrum pernix* | Q9YEG7 |
| *Sulfolobus islandicus* (SlaA) | F0NHT7 |
| *Sulfolobus islandicus* (SlaB) | F0NHT6 |
| *Metallosphaera sedula* (SlaA) | A4YHQ8 |
| *Metallosphaera sedula* (SlaB) | A4YHQ9 |
| *Nitrososphaera viennensis* (SlaA) | WP_144239589.1 |
| *Nitrososphaera viennensis* (SlaB) | WP_144239588.1 |

## Supplementary Movie Legend

**Movie S1. Atomic structure of the *D. radiodurans* S-layer.**

The cryo-EM map and atomic structure of the *D. radiodurans* S-layer show how the immunoglobulin-like domains of HPI form the lattice. Different views of the S-layer are shown with text annotations.

**Supplementary References**


1. Y. Z. Tan *et al.*, Addressing preferred specimen orientation in single-particle cryo-EM through tilting. *Nat Methods* **14**, 793-796 (2017).

2. K. Yamashita, C. M. Palmer, T. Burnley, G. N. Murshudov, Cryo-EM single-particle structure refinement and map calculation using Servalcat. *Acta Crystallogr D Struct Biol* **77**, 1282-1291 (2021).

3. J. Jumper *et al.*, Highly accurate protein structure prediction with AlphaFold. *Nature* **596**, 583-589 (2021).

4. J. Zivanov, T. Nakane, S. H. W. Scheres, Estimation of high-order aberrations and anisotropic magnification from cryo-EM data sets in RELION-3.1. *IUCrJ* **7**, 253-267 (2020).

5. T. Bepler *et al.*, Positive-unlabeled convolutional neural networks for particle picking in cryo-electron micrographs. *Nat Methods* **16**, 1153-1160 (2019).
